# 3D printing for an anterolateral thigh phalloplasty

**DOI:** 10.1186/s41205-023-00200-z

**Published:** 2023-12-19

**Authors:** Maxwell W. Walker, Christodoulos Kaoutzanis, Nicholas M. Jacobson

**Affiliations:** 1 School of Engineering, Computation, and Design – Inworks Innovation Initiative, University of Colorado Anschutz Medical Campus, Aurora, USA; 2 Anschutz Medical Campus; School of Medicine; Plastic Surgery, UCHealth University of Colorado Hospital, Aurora, USA

**Keywords:** 3D Printing, Vessel location, Anterolateral thigh (ALT) flap, Phalloplasty

## Abstract

**Background:**

Phalloplasty procedures are performed to create a phallus, typically as a gender-affirming surgery for treating gender dysphoria. Due to the controversial nature of this specific procedure, more innovation is needed to directly assist surgical teams in this field. As a result, surgeons are left to improvise and adapt tools created for other procedures to improve surgical outcomes. This study developed a patient-specific 3D printed model from segmented computed tomography (CT) scans to accurately represent the relevant vasculature necessary for anterolateral thigh (ALT) flap phalloplasty. The surgical procedure seeks to maintain intact vessels that derive from the descending branch of the lateral circumflex femoral artery, typically found traveling within the intermuscular septum between the rectus femoris and vastus lateralis.

**Methods:**

In this study, we created and printed 3D models of the leg and vasculature using two techniques: (1) a standard segmentation technique with the addition of a reference grid and (2) a bitmap method in which the total CT volume is colorized and printed.

**Results:**

The results gathered included the physician’s view on the model’s accuracy and visualization of relevant anatomy. Bitmap-printed models resulted in a high amount of detail, eliciting surgeons’ undesirable reactions due to the excess of information. The hybrid method produced favorable results, indicating positive feasibility.

**Conclusions:**

This study tested the ability to accurately print a patient-specific 3D model that could represent the vasculature necessary for ALT flap procedures and potentially be used in surgical reference and planning in the future. A surgeon performing phalloplasty procedures discussed their approval of both models and their preference for grid creation and application.

## Introduction

The number of individuals who identify as transgender is surging. In the United States, from 2017 to 2020, an estimated 1.3 million adults identified as transgender, with 35.9% of the 1.3 million identifying as transgender men [1]. Often, these individuals struggle with gender dysphoria, described as discomfort and stress due to a person’s physical or sexual features not matching their gender identity [2]. This dysphoria can lead to symptoms of depression and anxiety and an increased rate of self-harm and suicidal tendencies. Creating a phallus allows for a physical change of the external genitalia and can combat feelings of gender dysphoria [3]. As such, phalloplasty procedures are increasingly performed to construct a phallus as part of gender-affirming care. In fact, in a 2015 United States Transgender Survey (*n* = 27,715), the percentage of trans men who underwent phalloplasty procedures was 3%, with 19% stating they “want it someday [4].”

**Fig. 1:**
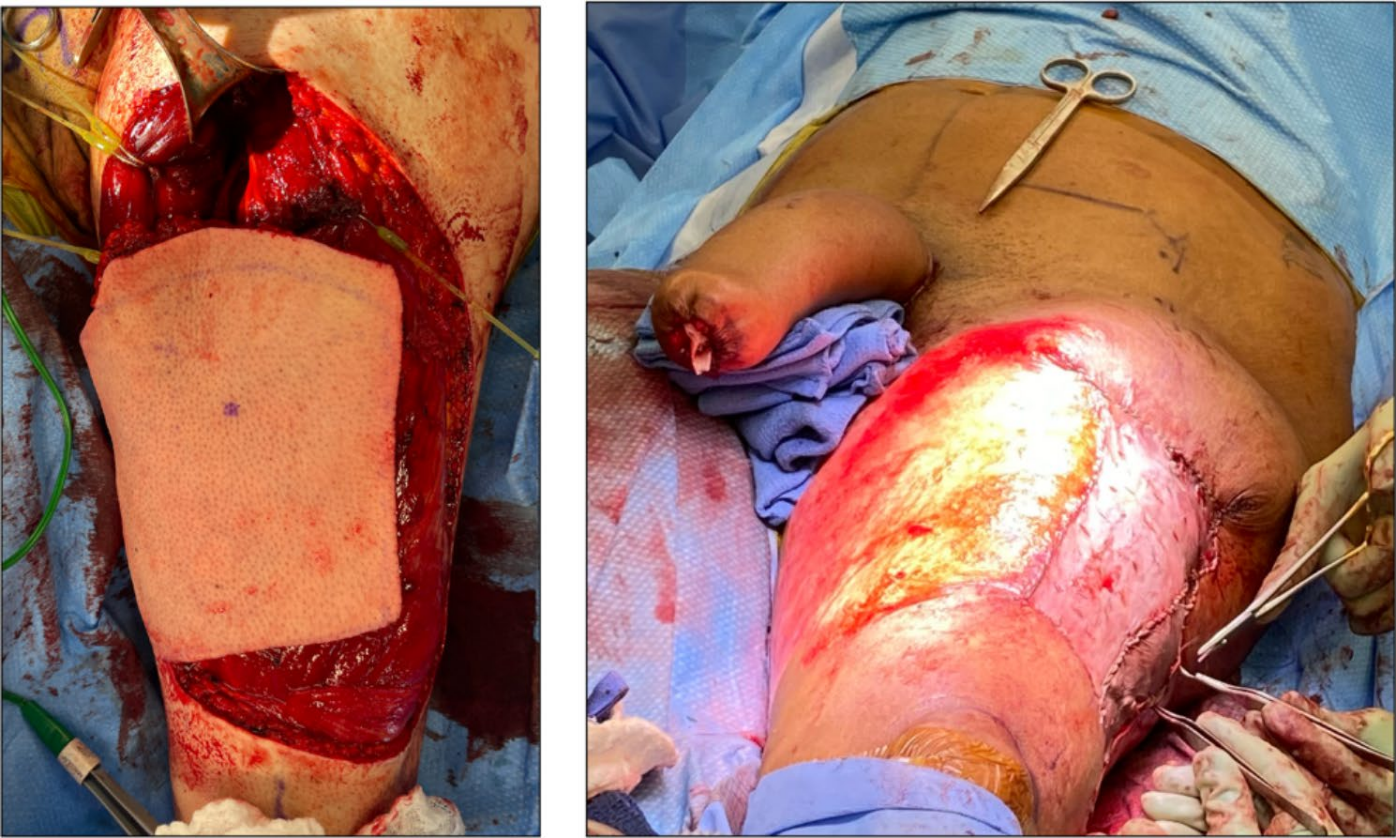
(Left) Harvested ALT Flap prior to being relocated through the tunnel indicated by the surgical retractor in the top of the image. (Right) Finished ALT phalloplasty procedure showing the constructed phallus, prior to the aesthetic and functional procedures to happen in the future. Additionally, this image shows the surgical team sewing a skin graft over the harvested area

Various flaps can be used when constructing a phallus, such as the radial forearm free flap and the latissimus dorsi musculocutaneous free flap. In free flap procedures, the flaps are removed from their respective location, and the vasculature is disconnected and then re-anastomosed to the recipient vessels once transplanted to the proper area. In contrast, for pedicle flaps, such as the anterolateral thigh (ALT) flap, the vasculature is not disconnected and only requires the sensory nerve of the thigh to be reattached. Specifically, the lateral femoral cutaneous nerve is coapted to one of the two clitoral nerves, allowing the potential sensation recovery to the newly constructed phallus. The pedicled ALT flap is harvested and passed under the rectus femoris muscle and sartorius muscle and through a subcutaneous tunnel between the thigh and the mons pubis area, where it is pulled out through an opening and inset to the area after it is shaped into a phallus (Fig. 1). The phallus will then be altered according to the patient’s preferences: e.g., to create the glans penis, scrotum, and to receive an implantable erectile prosthetic. The use of the ALT flap first began in 1984 during free flap surgeries of reconstruction in the head and neck area [4]. The ALT flap has a low donor morbidity rate and can be used as a pedicled flap, which could reach far from the original harvested site [5].

**Fig. 2:**
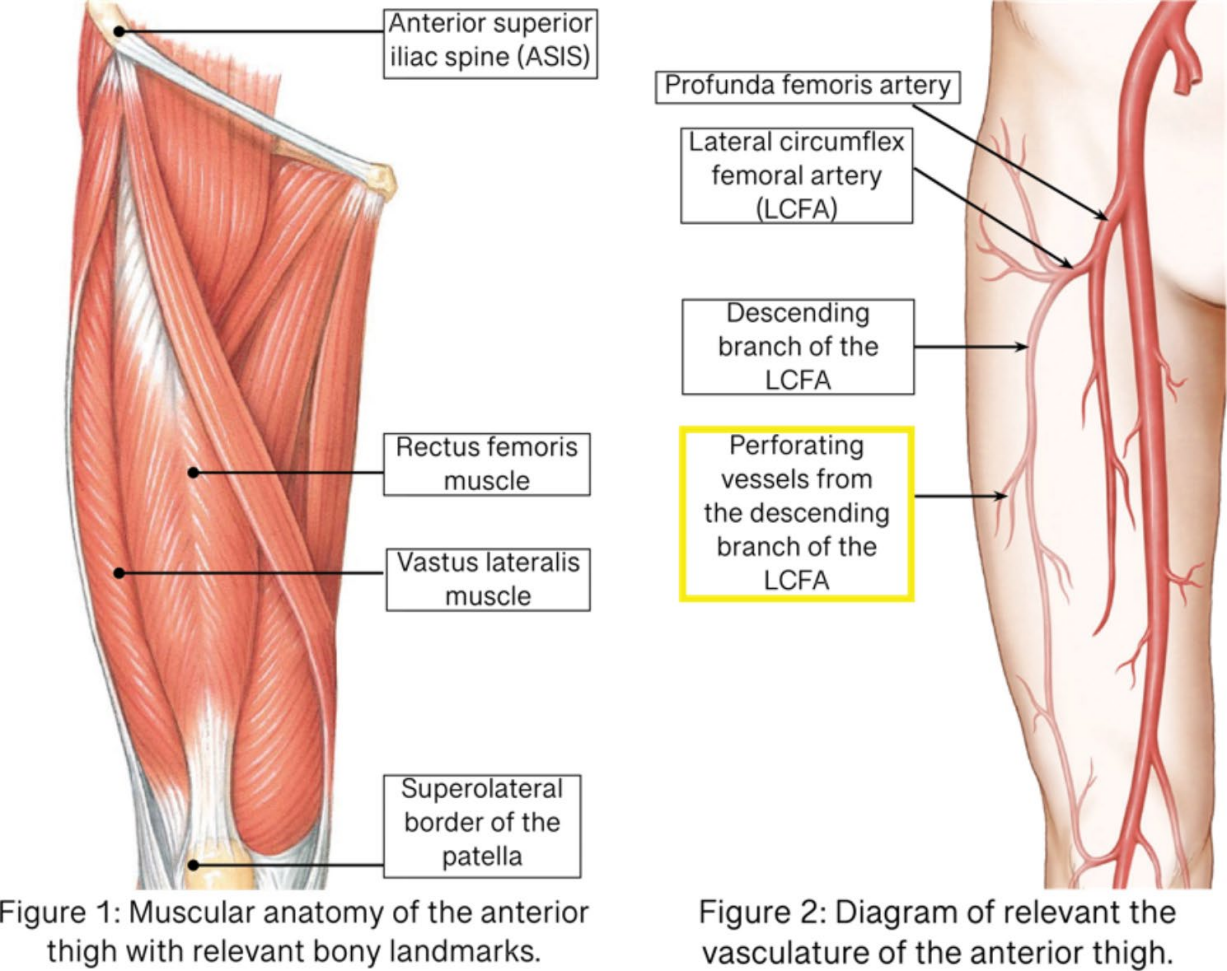
(Left) Relevant musculature of the anterior thigh & bony landmarks used during flap harvesting. (Right) Relevant vasculature of the anterolateral thigh supplying the ALT flap area

The vasculature most commonly sought-after during ALT flap harvesting is perforating vessels branching off the descending branch of the lateral circumflex femoral artery (LCFA) [6]. In roughly 90% of the cases, the descending branch of the LCFA can be found traveling within the intermuscular septum between the rectus femoris and vastus lateralis (see Fig. 2). However, additional perforators can also be located off the transverse branch of the LCFA or the femoral artery [7].

When finding the perforating vessels, current procedures typically rely on 2D patient computerized tomography (CT) scans and surface-level anatomical landmarks to find a midpoint between the anterior superior iliac spine (ASIS) and the superolateral border of the patella [8]. This landmark is the reference point for searching for the vessels deep into the skin. However, vasculature variability can complicate this approach and the complexities in projecting 2D structures on a 3D design.

Recent studies have shown that using three-dimensional anatomical models improves spatial recognition and visualization of vasculature during surgical procedures [9]. Moreover, using 3D models for surgical planning and anatomical reference has been shown to reduce surgical times and complications [10]. In this study, we adapted a methodology for deep inferior epigastric perforator (DIEP) flap surgeries to create an accurate patient-specific 3D printed model from CT scans [11]. This method could more accurately represent the relevant vasculature in the ALT flap and improve phalloplasty procedures by reducing overall surgery times and complications.

Current printed 3D patient anatomical models typically come from the mesh-based segmentation approach. This segmentation creates an isosurface through thresholding intensity values for specific anatomical structures. This process is then manually edited to refine further the isosurface, which is time-consuming and subject to human error. While automated methods for segmentation are available, they do not always account for anomalies or diseases and often require a manual editing step. A new form of 3D model creation lies in bitmap printing; in this format, models are created quicker and more complex as the models are printed directly from medical images with no segmentation involved. These models are “superior in spatial and contrast resolution to current 3D modeling methods” while allowing for “soft tissue differentiation” [12–14]. Both factors are essential for anatomical reference as they directly represent the patient scan data, which is crucial for surgical planning. With this novel printing methodology, much must be explored and refined to produce models with high clarity and consistency. Bitmap printing has the potential to innovate surgical planning as models are created quickly and with an in-depth accuracy of anatomical structures, such as small perforating vessels, that are difficult to perceive from a 2D patient scan.

This feasibility study is designed to test the hypothesis that CT data sets can be used to create an accurate 3D-printed model of the vasculature supplying the ALT flap. Designing models with our current procedural steps in mind, the goal was to create a tool that was easy to implement and did not interfere with the surgical planning process. The surgical team can understand and reference the model quickly and confidently by taking already-used landmarks. A model that needs to be simplified and easier to understand would disrupt surgical planning and is likely to be used by the surgical team. We tested this hypothesis by using contrasted CT datasets of the thigh and creating two types of 3D-printed models. The first model was created by segmenting the relevant anatomical structures from the CT DICOM data, and a grid was created and applied to the models for a measurable reference. The second model was created via bitmap printing in which intensity values within the scan are remapped to emphasize vasculature. Both models were then printed using a J5 Medijet printer.

**Fig. 3:**
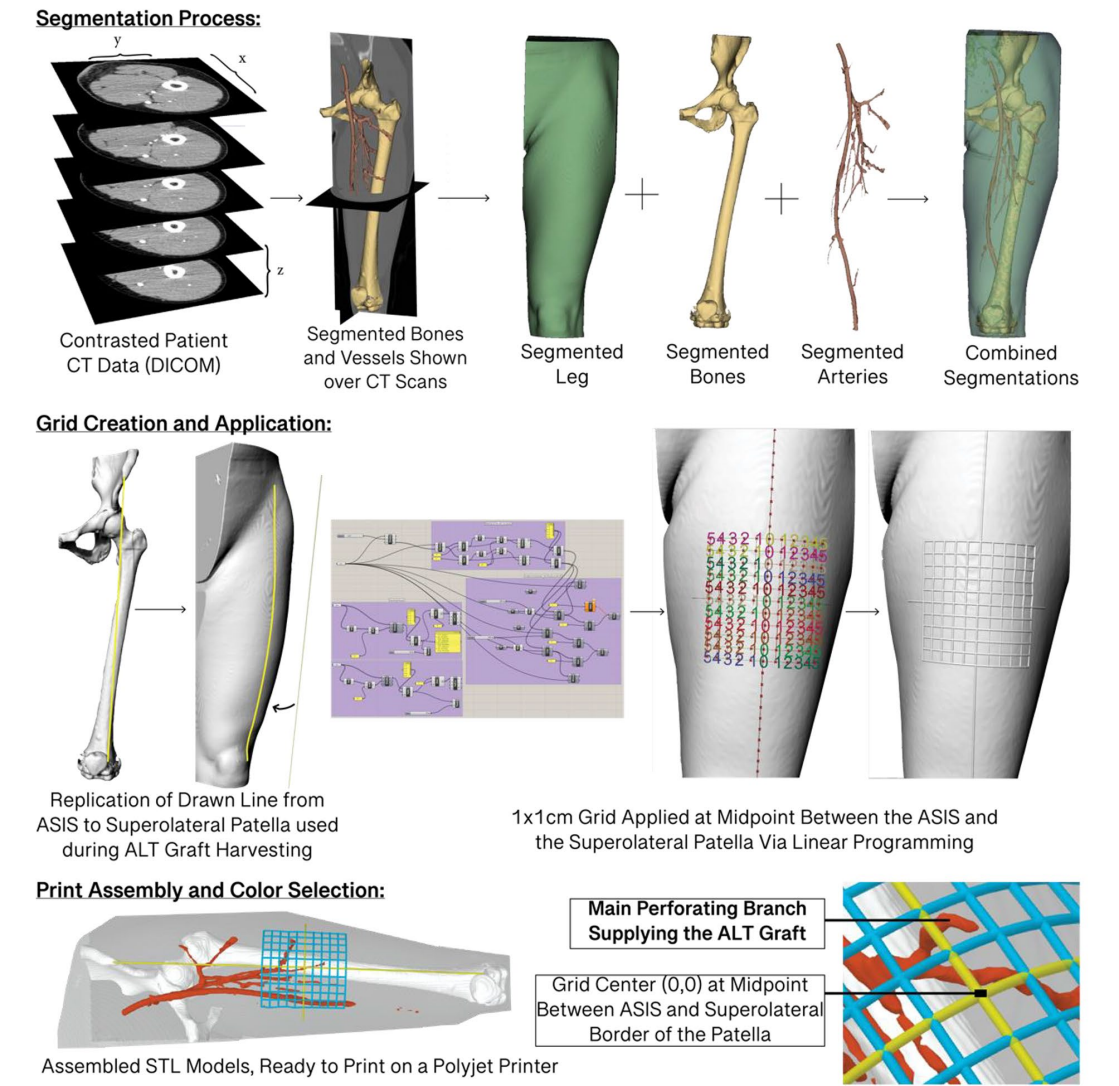
Workflow of the segmentation process, grid creation & application, and model preparation

Additionally, this study examined the comparison of STL and Bitmap-based models for use during surgical planning, how the two methods represented the necessary anatomy, and how easy the models were to understand. We hypothesized that the surgeon would favor the STL-based models due to the ability to isolate specific anatomy and include the reference grid, two features that are not attainable within the bitmap-based models. This hypothesis was evaluated based on the surgeon’s feedback on each model, specifically around how the models provide insight to execute a surgical plan compared to 2D CT DICOM data.

The accuracy of these models was evaluated by the surgical team performing phalloplasty procedures. Upon review, the surgical team evaluated the models to display the relevant vasculature necessary for ALT flap phalloplasty accurately. These results provide positive initial data to push this method forward to be potentially tested clinically on its ability to reduce operative time and complications, including failure rates.

**Fig. 4:**
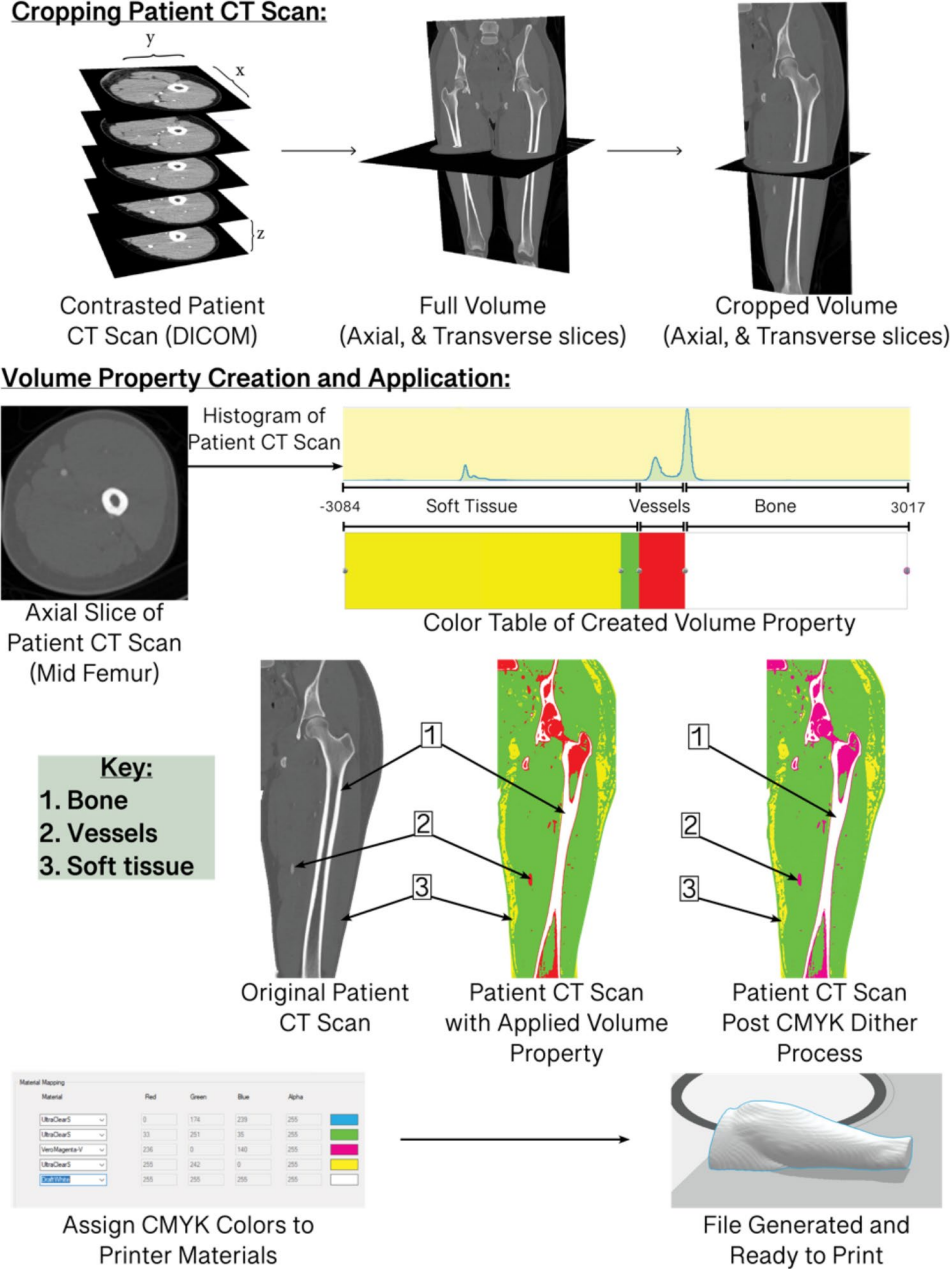
Workflow of Bitmap print creation from patient CT data

## Materials and methods

### Dataset sources

Deidentified CT scans were acquired from IDOIMAGING open-source databases. All scans utilized an axial resolution of 240 × 240 with a 4 mm slice thickness and 2 mm image spacing. These DICOM data sets were then uploaded into 3D Slicer 5.2.1 (2022) to be modeled and segmented.

### Creation of segmentation-based 3D models (Fig. 3)

Once uploaded into 3D Slicer, the relevant anatomical structures were segmented from the CT scans. The segmentations were made up of the bones (femur, pelvis, patella), the total volume of the thigh, the intermuscular septum between the rectus femoris and vastus lateralis, and the vasculature supplying the ALT flap area. The goal is to combine the structures to preserve the relation of the vessels with the surrounding bone and soft tissue [11]. These segmentation models are then exported into Rhinoceros 3D 5.0, a surface-based modeling program, where a bounding box is created around the models, and the inferior-medial point of the box is moved to (0,0,0). Then, considering the current surgical method of locating vasculature using the midpoint of a line drawn on the skin from the ASIS to the lateral border of the patella, a line is drawn and projected onto the surface of the model to replicate this step in the surgical process digitally. Then, via linear programming in Photoshceros 3D 5.0 Grasshopper, the midpoint of this line is found, and a 10 × 10 cm grid is created and applied with (0,0) of the grid fixed at said midpoint. This grid remains accurate when wrapped around a curved surface and can be scaled down with the models to allow correct surgical reference. Then, the models are uploaded into Autodesk Monolith v.0.3 (2016), a bitmap-based modeling environment to adjust the internal fill of the models and export the files into Standard Tessellation (STL) Format. The STL files will be assembled, assigned colors/opacity values, and ready to 3D print using GrabCad (2022). Within GrabCad, the individual STLs are printed using a priority-based selection in which the various models are assigned a priority level. As the two models overlap, the one with higher priority will be printed in that location. In this study, the vessels were printed with the highest priority, followed by the grid lines, the bone, the intermuscular septum, and the entire leg volume. The models are printed on the Stratasys J5 Medijet multi-color polyjet printer, processed, cleaned, and clear coated to increase resolution and clarity.

### Creation of Bitmap-Based 3D Models (Fig. 4)

The second method this study examines is bitmap-based printing’s ability to represent the relevant anatomy in the anterolateral thigh. A volume of bitmaps, or 3D pixels, can represent various structures, manipulated in color, and then printed in a 3D model. For this method, the CT scans were uploaded into the bitmap-based software 3D slicer (2022), and the volume was cropped only to include the thigh. Then, a volume property was created in which specific colors were applied to different intensity values within the scan. These intensity values represent various anatomical structures in the CT scans, such as bone, soft tissue, and vessels. With the application of the volume property, a bitmap was generated by virtually slicing the model and exporting the slices into a folder for the next step. These slices were imported through Adobe Photoshop 2023. They were dithered, and their colors were adjusted from multiple shades and hues into approximated cyan, magenta, yellow, and white colors. The slices were then uploaded into Grabcad print’s bitmap printing app, creating a “Grabcad bitmap file”(.gcvf). Creating this file format allowed for the four colors in the slices to be assigned to colors available in the printer. For this study, cyan, green, and yellow were assigned to clear, magenta to magenta, and white to white. Once the models were ready, they were printed on a Stratasys J5 Medijet printer.

### Post processing of models

Once the prints were finished, they were sanded and sprayed with industrial acrylic lacquer paint to improve the appearance and visibility of the models.

**Fig. 5:**
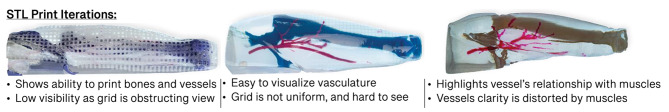
STL print iterations with positive and negative feedback for each model

### Evaluation of accuracy

The 3D-printed models were then compared to the 2D CT images segmented from them. These models’ general appearance and accuracy were then assessed by consulting the plastic surgery team on the accuracy of vessel location and resemblance to previous surgical cases based on the experience of having performed numerous such procedures per year.

## Results

In this study, the results gathered included the physician’s view on the model’s accuracy and visualization of relevant anatomy. Both factors are essential for the model’s progression into a clinical trial. Below are the details of the resulting models and evaluations.

### STL-Based model of the thigh

The first method we explored was creating a model of the relevant anatomy in the leg via segmentation. We expected that the segmentation models would translate into a model that was easy to reference while remaining accurate. Upon review from the practicing surgeon, it was their opinion that the models accurately depicted the necessary anatomy. Additionally, the surgeon provided positive feedback on the creation and application of the reference grid with current surgical steps in mind. With multiple iterations of models (Fig. 5), the surgeon provided positive and negative feedback for each, as well as feedback on how the models could be adjusted and combined.

**Fig. 6:**
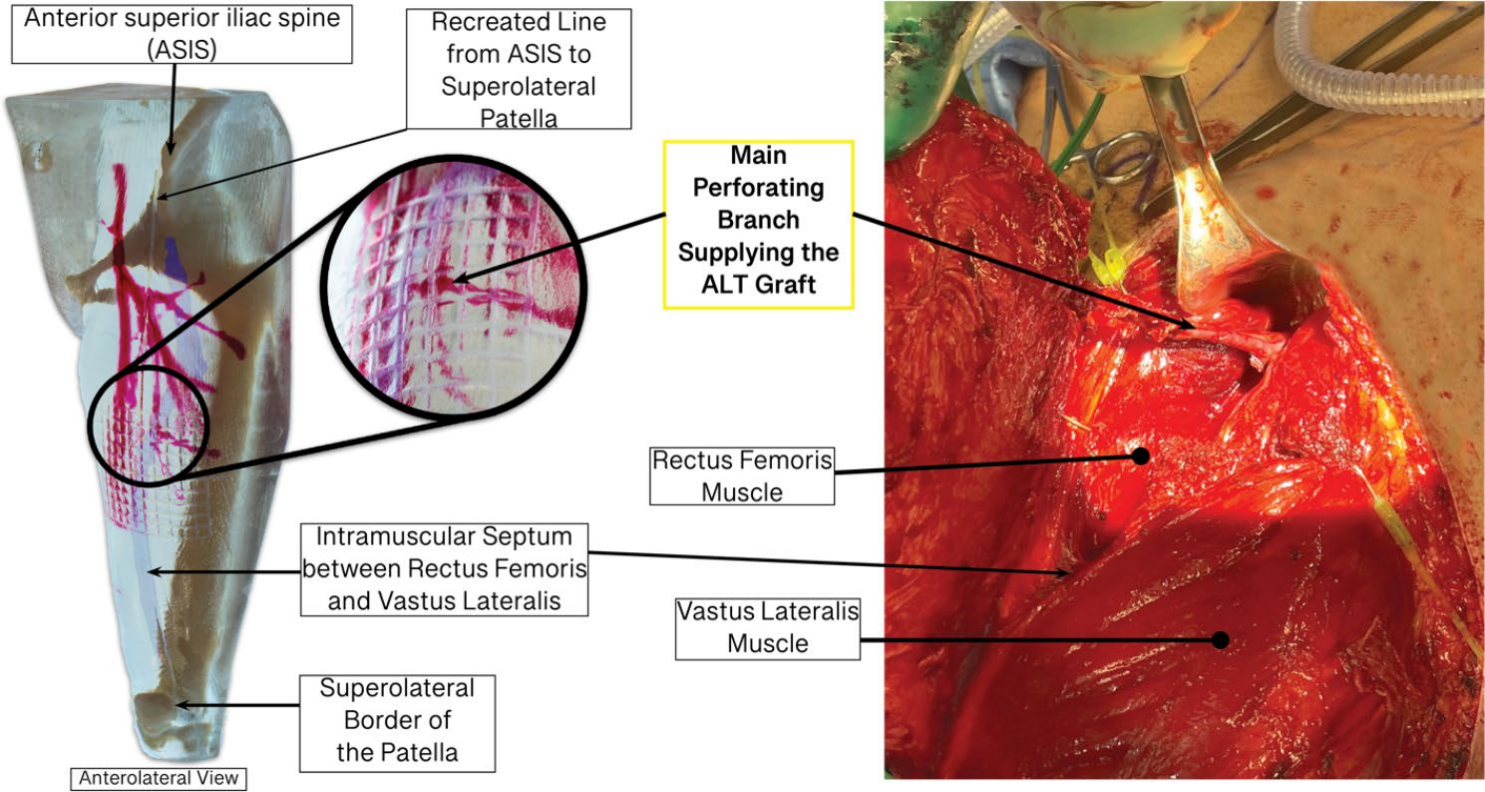
An anterolateral view of the most recent print iteration (left) with important structures and markings highlighted compared to a photo of the in-situ vasculature (right) that the model was created from

As expressed, the surgeon reacted positively to this model and disclosed their confidence in bringing the modeling into a surgical setting. They discussed how this model could be used in surgical planning and to improve surgical decision-making. This model includes the segmented anatomy, a reference grid applied to the already used midpoint, and the intermuscular septum between the rectus femoris and vastus lateralis for reference. All these features are included within a 3D model printed at a 30% scale of the original DICOM dataset. The most recent model, compared to living vasculature, is shown below. (Fig. 6)

**Fig. 7:**
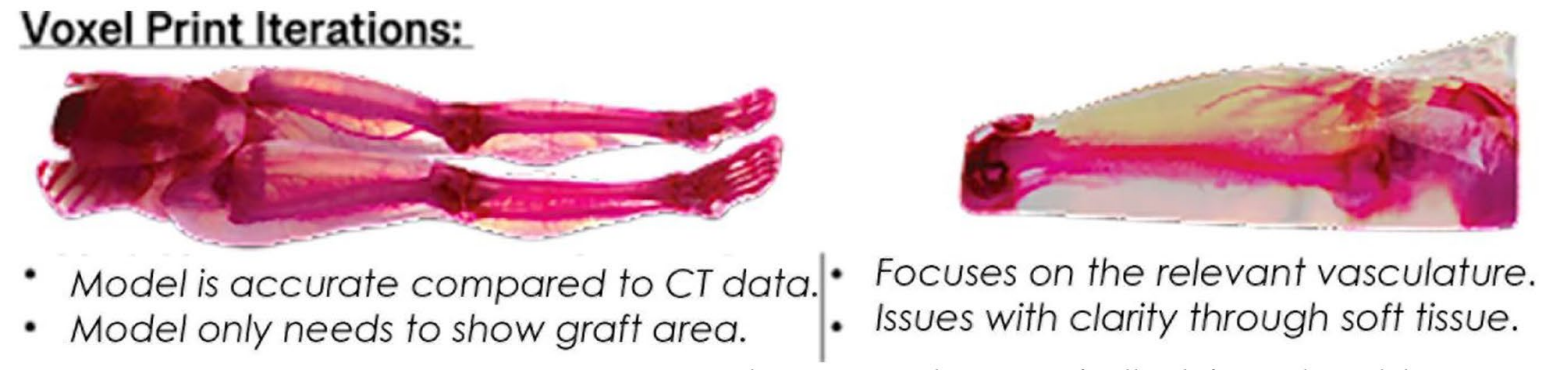
Bitmap print iterations with positive and negative feedback for each model

### Bitmap based model of the thigh

Like the STL-based model, the practicing surgeon was consulted on the accuracy and useability of the Bitmap-based model. With the creation of the bitmap-based model, we expected an accurate model that could show more detailed vasculature compared to the segmented model. This model highlighted the secondary and tertiary branches of vessels supplying the flap area while remaining accurate. This method also underwent several iterations to determine the best printing process and workflow (Fig. 7).

**Fig. 8:**
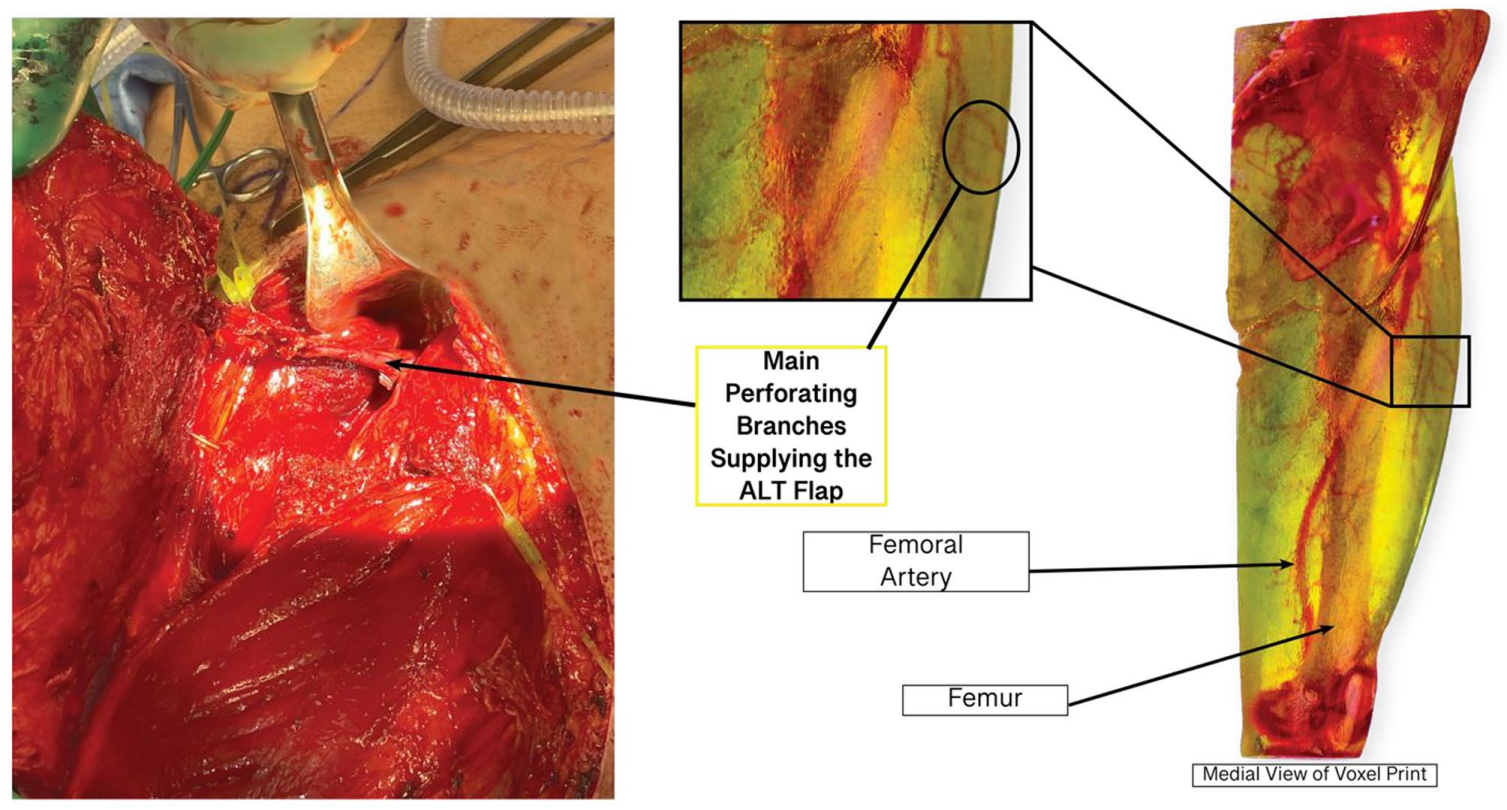
Most recent bitmap print compared to in-situ vasculature with essential structures and vasculature labeled

In a comparison between the two models, the bitmap-based model displayed more vasculature than the hand-segmented models. While it did not have a reference grid, it displayed a higher volume of supplying vessels in the ALT flap area. One concern the surgeon did discuss is if the model displayed ?too much? of the surrounding vasculature and could be confusing during surgical planning/reference. The vasculature remained accurate with the most recent model (Fig. 8) and can be seen below compared to an in-situ photo of the vasculature.

## Discussion

This study tested the ability to accurately print a patient-specific 3D model that could represent the vasculature necessary for ALT flap procedures and potentially be used in surgical reference and planning in the future. We create models through two methods, with both being evaluated as accurate. The surgeon performing the phalloplasty procedures discussed their approval of the models as well as their approval of the grid creation and application. Creating a patient-specific model that yields accurate results is crucial for the surgical team and just as necessary for the patient. With a surgical reference, the surgeons can approach the surgery more confidently, potentially reducing operative time, surgical obstacles, and unexpected findings. The ability to 3D model the vasculature supplying the ALT flap area, has been studied but focused on which type of scan yielded the best results [14]. Our study aimed to take one step further and create a tangible 3D model with a measurement reference that could increase the accuracy and confidence of the surgical team.

A 3D model created with a similar method has been tested in DIEP flap surgeries and yielded decreased operative times and lower surgical risk [15]. With the benefits of 3D models and an increase in industry attempting to create anatomical visualization methods, this method holds promise that it could be viable for use in other ALT flap-based procedures. Surgeons would benefit from a 3D guide of the respective bones, vessels, and soft tissue, and patients could benefit from a better-informed surgical approach. Additionally, these models can serve as clinical education tools for the residents and fellows as they learn to perfect their approach to ALT flap harvesting.

**Fig. 9:**
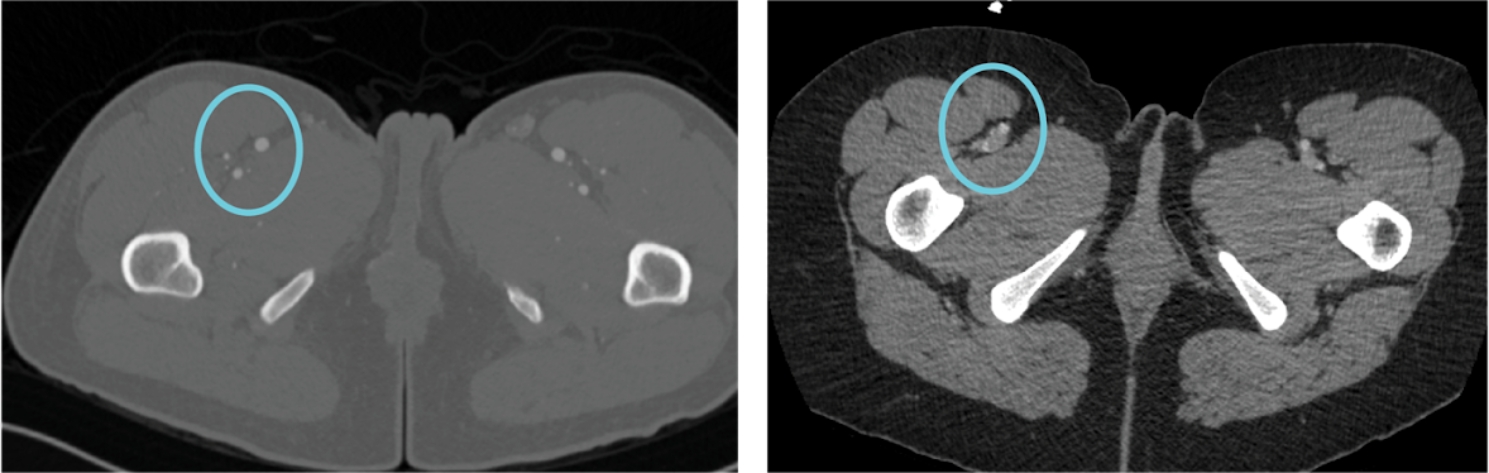
Comparison of contrasted CT Angio scan (Left) and non-contrasted CT-Scan (Right), with area inside circles displaying vasculature and their differences to the surrounding soft tissue

Two methods of 3D printing were tested during this study, and feedback on their respective positives and negatives became apparent with their evaluation. STL models allow for selecting which anatomical structures to highlight and omit from the models. While this decreases the model’s complexity, it can also be beneficial as it allows for isolating desired structures [16]. However, in the same regard, it can omit structures that may be important for surgical planning and execution. Also, within an STL-based model, there is more freedom for size, color, and opacity alterations during the creation process. With bitmap-based printing, the prints directly represent the CT volume; thus, the anatomy is complex and accurate for patient scans.

In some cases, this complexity may provide too much information, making the model more explicit, as extraneous anatomy can obscure essential structures. Additionally, a lack of adjustments can be made during the creation process within the bitmap printing process. The Bitmap-based models are only fully perceived once they are printed and processed; this can cause issues as any issues must be addressed after the print is finished rather than during the process.

While this study was evaluated to yield accurate results, it possessed various limiting factors. The main limiting factor for this study was a low patient population. While this method was considered accurate through visual inspection, it must be tested on a broader range of cases. The lack of access to healthcare and ongoing political debate over the rights of trans-identifying individuals bring even more obstacles to supplying a consistent and large sample size. Another limiting factor in this study was the quality of CT scans. Some imaging data was deemed unusable for model creation as the quality could have been better, or the scans needed portions related to the surgery. In this study, patients with CT angiography scans had the best ability to differentiate the vessels from the surrounding soft tissue in both models. (Fig. 9).

With improved and consistent model creation, this method can be turned into a clinical trial to test the model’s effect on flap harvesting duration, surgical team confidence, and overall surgical outcomes. In a clinical trial, the demographic must include phalloplasty patients who have received CT angiography scans within the six months before their procedure. These models will also be assessed regarding their ability to be combined/used together in a surgical setting. Additional studies will be performed on optimizing scans for the best-quality prints.

## Data Availability

All data will be provided by the author upon request. Nicholas.Jacobson@Cuanschutz.edu.
